# Low-Cost, Disposable, Flexible and Highly Reproducible Screen Printed SERS Substrates for the Detection of Various Chemicals

**DOI:** 10.1038/srep10208

**Published:** 2015-05-14

**Authors:** Wei Wu, Li Liu, Zhigao Dai, Juhua Liu, Shuanglei Yang, Li Zhou, Xiangheng Xiao, Changzhong Jiang, Vellaisamy A.L. Roy

**Affiliations:** 1 Laboratory of Printable Functional Nanomaterials and Printed Electronics, School of Printing and Packaging, Wuhan University, Wuhan 430072, P. R. China; 2 Department of Physics and Materials Science, City University of Hong Kong, Tat Chee Avenue, Kowloon Tong, Hong Kong SAR, P. R. China; 3 Key Laboratory of Artificial Micro- and Nano-structures of Ministry of Education, School of Physics and Technology, Wuhan University, Wuhan 430072, P. R. China; 4 State Key Laboratory for Powder Metallurgy, Central South University, Changsha 410083, P. R. China

## Abstract

Ideal SERS substrates for sensing applications should exhibit strong signal enhancement, generate a reproducible and uniform response, and should be able to fabricate in large-scale and low-cost. Herein, we demonstrate low-cost, highly sensitive, disposable and reproducible SERS substrates by means of screen printing Ag nanoparticles (NPs) on a plastic PET (Polyethylene terephthalate) substrates. While there are many complex methods for the fabrication of SERS substrates, screen printing is suitable for large-area fabrication and overcomes the uneven radial distribution. Using as-printed Ag substrates as the SERS platform, detection of various commonly known chemicals have been done. The SERS detection limit of Rhodamine 6G (R6G) is higher than the concentration of 1 × 10^−10^ M. The relative standard deviation (RSD) value for 784 points on the detection of R6G and Malachite green (MG) is less than 20% revealing a homogeneous SERS distribution and high reproducibility. Moreover, melamine (MA) is detected in fresh liquid-milk without additional pretreatment, which may accelerate the application of rapid on-line detection of MA in liquid milk. Our screen printing method highlights the use of large-scale printing strategies for the fabrication of well-defined functional nanostructures with applications well beyond the field of SERS sensing.

SERS is a powerful vibrational spectroscopy technique that allows for highly sensitive structural detection of low-concentration analytes via the amplification of electromagnetic fields generated through the excitation of localized surface plasmon resonances (LSPR) of the substrate^1^. Consequently, SERS is crucially dependent upon the substrate, where excitation of the localized metal surface plasmon resonance enhances the vibrational scattering signal of proximate analyte molecules^2^. Several years of research have been devoted for creating and optimizing SERS substrates in order to provide the largest enhancements possible^3^,^4^,^5^.

The current fabrication methods (such as electrochemically roughened electrodes, lithography-based microfabricated substrates, colloidal self-assembly) for SERS substrates are often complex and time-consuming. As a competitive alternative, the development of simple, facile and low-cost methods are becoming increasingly important^6^. Printing technology is a strong candidate for high throughput, facile and cost-effective fabrication of large-scale orderly functional patterns or arrays. It is purely an additive method in which an ink is added depending on the necessity. Therefore, patterns can be formed in a single step. For example, Yu and White have reported a low-cost SERS substrate by modifying the surface chemistry of cellulose paper with patterned nanoparticle arrays, all with a consumer inkjet printer^7^. Qu and co-workers have reported high-sensitive screen printed SERS substrates with excellent SERS activity under optimal fabrication conditions^8^. Therefore, the advantages of printing are the capability in the areas of mass production and quality control over the deposition area and thickness. In addition, the ink can be printed on a wide variety of substrates, including paper, fabrics, plastics, etc.^9^,^10^, which are vital for the advent of practical application and also a widespread industrially-applied method. For example, with a pitch of printed lines as fine as 250 μm, the printing process can significantly reduce the time and cost associated with photolithography^11^. Moreover, it is simple, affordable and adaptable with various preparation conditions. The screen printing technique is often employed to manufacture the conductive lines and electrodes in large scale, but its application to the fabrication of flexible and highly reproducible SERS substrates or arrays are scarce so far. Currently, the poor reproducibility of SERS signal becomes the major obstacle for the development of SERS field.

Therefore, efforts have been devoted to solve the aforementioned problem. On this regard, herein, we present a large-scale fabrication method to manufacture low-cost, disposable, flexible and highly reproducible SERS array substrates by employing a high-throughput screen printing method. The SERS performance for various chemicals, including R6G, MG, 4-aminothiophenol (ATP), 2-Mercapto-5-nitrobenzimidazole (MNB), Coomassie Brilliant Blue R-250 (BBR) and melamine in fresh milk have been systematically investigated. The as-printed dot arrays exhibit an excellent SERS performance, especially the signal reproducibility and detectability for various chemicals. In comparison with the aforementioned references^7^,^8^, our screen printed Ag patterns show high signal reproducibility for different analytes. A RSD value of less than 20% for more than 700 points for R6G, MG and MA in fresh milk has been obtained. The results reveal that the as-printed SERS dot arrays can be used for SERS substrates.

## Method Sections

### Materials

Glucose, silver nitrate (AgNO_3_), polyvinylpyrrolidone (PVP), sodium carbonate (Na_2_CO_3_), ethanol, Rhodamine 6G (R6G), Malachite green (MG), 4-aminothiophenol (ATP), 2-Mercapto-5-nitrobenzimidazole (MNB), Coomassie Brilliant Blue R-250 (BBR) and melamine (MA) were purchased from Sigma-Aldrich. All chemicals were used as received without further purification.

### Synthesis of Ag nanoparticle inks

The silver NPs were synthesized by the reduction of glucose in the presence of PVP as capping agent. 0.003 mol AgNO_3_, 0.0015 mol glucose, 1 g PVP and a 0.0022 g sodium carbonate were added in a sequence in water under stirring at room temperature, and then heated to reaction temperature. A 0.3 ml of triethylamine was dropped into the above mixtures by a peristaltic pump (0.6 ml/min), and the color of the reaction solution changed rapidly. Finally, the resulting dispersion was washed thrice with ethanol at 10000 rpm for 10 min via centrifugation. As synthesized silver NPs were dispersed in ethanol at a concentration of 30wt%.

### Screen printing of Ag inks

In flat screen printing process, the as-obtained Ag ink was forced through the nylon coved screen printing plate. For printing the SERS substrates, the fineness of fabrics is 350 fiber/cm. As-obtained Ag ink was transferred through the patterned apertures and deposited onto the PET substrate by movement of a squeegee.

### Characterization

Transmission electron microscopy (TEM), high-resolution TEM (HRTEM), and energy-dispersive X-ray spectroscopy (EDX) were carried out using a JEOL JEM-2100F transmission electron microscope at 200 kV. The samples were dissolved in water and dropped on copper grids for inspection. UV-Vis measurements were conducted on a Shimadzu 2550 spectrophotometer. Field emission scanning electron microscopy (FSEM) studies were performed on a FEI Nova 400 NanoSEM operated at 20 keV. X-ray diffraction (XRD) patterns of the samples were recorded on a D8 Advance X-ray diffractometer (Germany) using Cu Kα radiation (λ = 0.1542 nm) operated at 40 kV and 40 mA and at a scan rate of 0.05° 2θ s^−1^. The Raman measurements for the samples were performed by a laser confocal micro-Raman spectrometer (RenishawinVia-Renishaw, 532 nm and 514 nm excitation wavelength, the diameter of laser spot size is around 700 nm at 100x magnification, max laser power is 50 mW with the exposure time 1 s, and the applied power was set to 0.01% for all the spectra). An amount of 10 μl solution (chemicals dissolve in ethanol) was dropped on the surface of printed Ag pattern for SERS measurements, and dried before measurements. All spectra were calibrated with the silicon Raman mode at 520.7 cm^−1^.

## Results and Discussion

**Figure 1:**
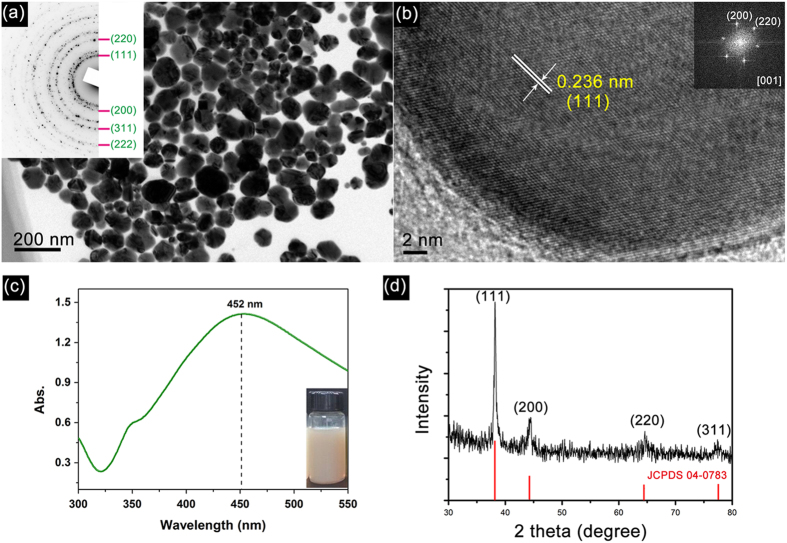
TEM image (**a**, insert is the corresponding SAED pattern), HRTEM image (**b**), UV-vis absorption spectrum (**c**, the insert is the photo of the inks) and XRD patterns (**d**) of as-prepared Ag NPs.

As shown in TEM images in Fig. 1a, as-obtained Ag NPs are composed of spherical particles with a mean diameter of 68.4 nm (statistical size histogram is present in Fig. S1 of *Support Information*). And the corresponding selected area electron diffraction (SAED) pattern shows that as-obtained Ag NPs have face centered cubic (*fcc*) crystallographic structure. The different diffraction planes are indexed as shown in the insert of Fig. 1a. The high-resolution TEM (HRTEM) image is shown in Fig. 1b revealing the lattice spacing of the Ag nanocrystal is 0.236 nm that corresponds to the (111) plane of Ag. More interestingly, most of the spherical Ag NPs have no structural defects and are single-entry crystals. The corresponding Fast Fourier Transforms (FFT) patterns (see the insets in Fig. 1b) show the generation of hexagonal diffraction pattern demonstrating that as-prepared Ag NPs are single crystals. In addition, some NPs clearly exhibit platelet structures with a preferential growth occurring on the (111) plane as shown in Fig. 1a. Surface plasmon bands appearing in the visible region are the characteristics of noble metal NPs. Ag NPs are often present a strong SPR absorption peak at ca. 400 -500 nm depending on its size, shape, and dielectric properties of the surrounding media^12^. As shown in Fig. 1c, the broad absorption bands at about 452 nm are considered to be due to surface plasmon resonance absorption of the electrons in the conduction bands of silver. The color of the Ag ink solution is dark yellow (see the insert photo). It is worth noting that the colloidal Ag NPs are found to be stable with no sedimentation for a month, and also no shift in SPR absorption peak is observed. Figure 1d shows the XRD pattern of the as-prepared Ag NPs in which four clear diffraction peaks are observed and indexed to the (111), (200), (220) and (311) planes of the *fcc* Ag. The XRD pattern further confirms the generation of Ag NPs and it is crystalline in nature.

**Figure 2:**
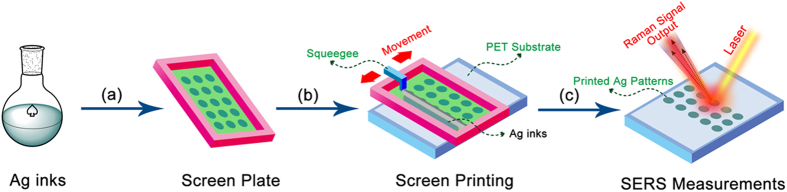
Schematic illustration of the fabrication process for large-scale SERS dot array substrates by screen printing: (**a**) preparing the Ag nanoparticle inks; (**b**) printing of Ag inks as dot arrays on PET by a designed scree plate; (**c**) dried and printed dot arrays for the determination of various chemicals via SERS techniques.

Screen printing is a fantastic artistic technique which is especially useful for printing on various substrates, and the process is easy, versatile and relatively cheap. In this work, screen plate is made up of a piece of porous, finely nylon mesh stretched over an aluminum frame. As shown in Fig. 2, the screen is placed over a PET substrate, and Ag ink is placed on top of the screen, and a filling bar is used to fill the screen with ink. The movement of squeegee with a pressure can let the Ag inks through the pattern area and appear on the PET. And then we obtain the designed Ag dot arrays on the PET substrate to determine various chemicals by SERS technique.

**Figure 3:**
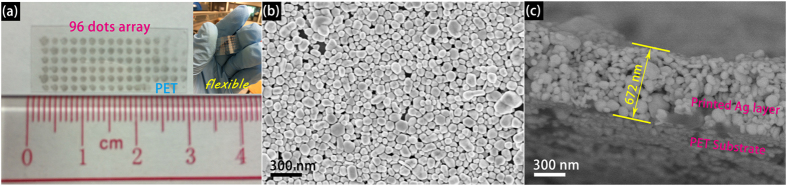
Photograph of the screen printed dots array substrate with dimensions of 15 mm × 30 mm including 96 dots (**a**) top-view (**b**) and cross-view (**c**) SEM image of the Ag layer from one dot.

Figure 3a shows the photograph of the screen printed dots array substrate, the circles of printed Ag NPs are designed to be 1 mm in diameter. During the printing process, the silver NPs appear brown on the PET initially and progressively become dark in color. Upon drying at 80 °C, the dots appear metallic gold yellow (see the insert photo). As is obvious from the insert of Fig. 3a, there is no chipping of Ag paint after bending or rolling the substrate, which demonstrate that as-printed Ag patterns are flexible and mechanically robust. This feature implies possible application of the product in flexible electronics. Figure 3b shows the top-view SEM image of the surface of printed dots with small and large particles. There is no aggregation and a homogeneous dispersion is seen over the large area. Clearly, the surface roughness and nanometer scale structure of printed Ag films is beneficial to the SERS measurements^13^. The cross-view SEM image is used to determine the thickness of the as-printed Ag film, as shown in Fig. 3c, the thickness is about 672 nm. If each dot is cylindrical, through estimation each dot requires an amount of 5.5 × 10^−7^ g of Ag, making it as low cost SERS detection technique.

**Figure 4:**
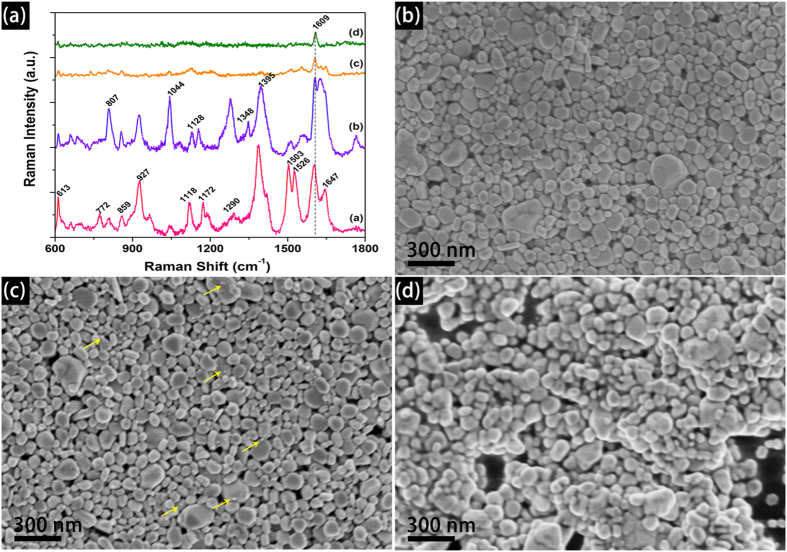
The SERS performance of the printed patterns under different drying temperature for R6G detection (1 × 10^−6^ M, the intensity have been normalized, the laser excitation is 532  nm) (**a**) and the representative SEM images of the samples dried at 120 °C (**b**) 160 °C (**c**) and 200 °C (**d**) respectively.

Because the printed patterns need to be dried and the effect of drying temperature (T_d_) on SERS performance has been analyzed. For R6G dyes, various SERS vibration modes can be found because R6G is nearly a planar molecule containing xanthene ring^14^. The curve **a** in Fig. 4a shows the representative feature peaks of R6G. While these SERS peak intensity (SPI) and location is different, all the peaks can be indexed to vibrational bands of R6G^15^. As shown in Fig. 4a, the normalized SPI of as-printed Ag dots under different drying temperature is different. Among the four samples, a strongest SPI is observed for the drying temperature of T_d_ = 80 °C, followed by the samples T_d_ = 120 °C and T_d_ = 160 °C, and the sample dried at T_d_ = 200 °C possesses the weakest SPI. The surface morphology as well as the average size of the silver particles are proved to be important in realizing high-performance SERS detection. Many studies reveal that the diameter of Ag NPs for acquiring optimal SERS effect is in the range of 20 to 70 nm^16^,^17^. Therefore, we use SEM to observe the surface morphology of the substrates at different temperature, and the results are shown in Fig. 4b–d. In comparison with Fig. 3b, the change of surface morphology for T_d_ = 120 °C is not clear. When the T_d_ is elevated to 160 °C, many small Ag grains are segregated from Ag NPs (arrows labeled in Fig. 4c). If the T_d_ is elevated to 200 °C, the Ag NPs are sintered and welded together. The above results reveal that higher drying temperature is not beneficial for SERS application.

**Figure 5:**
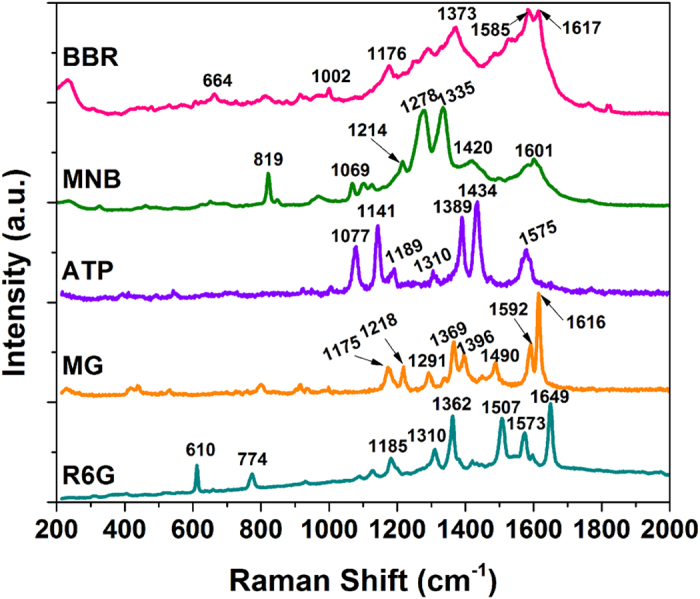
Arithmetically averaged SERS performance of the screen printed patterns for R6G (1 × 10^−6^ M), Malachite green (MG, 1 × 10^−6^ M), 4-aminothiophenol (ATP, 1 × 10^−3^ M), 2-Mercapto-5-nitrobenzimidazole (MNB, 1 × 10^−4^ M) and Brilliant Blue R (BBR, 1 × 10^−5^ M) (all the chemicals is dissolved in ethanol and the laser excitation is at 514 nm).

Owing to the best SERS performance of the sample with T_d_ = 80 °C, we used as-obtained dot array under this parameter to detect various chemicals, which are often used in different field, and the results are shown in Fig. 5. To ensure a better match between the localized surface plasmon resonance peak position of Ag nanoparticle of 452 nm, the incident light of laser was tuned from 532 to 514 nm. Among the five chemicals, R6G is often used as a tracer dye within water to determine the rate and direction of flow and transport, and also used extensively for biotechnology applications. MG is a carcinogenic and mutagenic agent and banned in many countries, such as the European Union, USA and China^18^. ATP is a common Raman active probe for obtaining the finger print SERS signal^19^. MNB is often used to treat electrodes for reducing the contact resistance and significantly improve the field-effect mobility in electronics industry, but MNB is an irritant. BBR is commonly used for the detection of proteins in biotechnology. We use the screen printed Ag patterns to detect the SERS spectra of the above mentioned five chemicals, the characteristic bands of these chemicals are all clearly observed and their corresponding vibration modes are depicted in Table 1. The above results reveal that as-printed Ag patterns are effective to detect various chemicals by screen printed SERS substrates.

**Figure 6:**
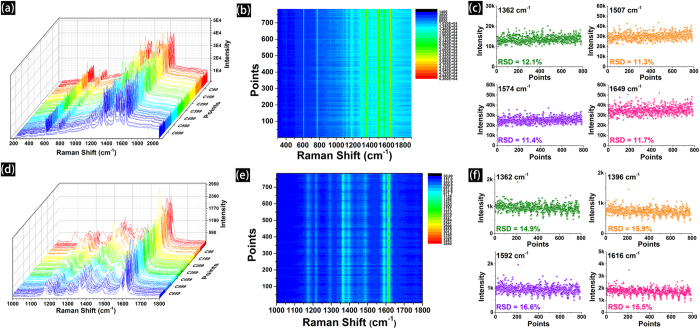
Raman waterfall plots, contour plots and RSD (the result was statistically analyzed from the original intensity of every predominate peaks) of different predominant bands conveying both the intensity and reproducibility of 784 points (a randomly selected area, the integration time was 2 s, step width is 3 μm) from the screen printed Ag patterns for R6G (1 × 10^−6^ M, **a**, **b**, **c**) and MG (1 × 10^−6^ M, **d**, **e**, **f**).

A good SERS substrate exhibits not only high enhancement ability but also good reproducibility^20^. To evaluate the reproducibility of the screen printed Ag patterns, we use time evolution SERS spectra to monitor the signal output and study the reproducibility of the screen printed Ag patterns for R6G and MG with a concentration of 1 × 10^−6^ M. As shown in Fig. 6a, Raman waterfall plots of R6G (1 × 10^−6^ M) recorded from a randomly selected area of the screen printed Ag pattern (784 signals). Figure 6b shows the corresponding contour plots of every Raman peaks with the excitation lines at 610, 774, 1185, 1362, 1507 and 1649 cm^−1^. These results reveal that the intensity and reproducibility of 784 points are stable and uniform. According to the statistics of predominant peaks’ intensity, the relative standard deviation (RSD) for the band vibrations of R6G at 1362, 1507, 1574 and 1649 cm^−1^ are 12.1%, 11.3%, 11.4% and 11.7%, respectively (Fig. 6c). Figure 6d,e shows the Raman waterfall plots and corresponding contour plots of 784 points, and the excitation line is at 1175, 1218, 1291, 1362, 1396, 1592 and 1616 cm^−1^. And Fig. 6f shows the RSD for the band vibrations of MG at 1362, 1396, 1592 and 1616 cm^−1^ are 14.9%, 15.9%, 16.6% and 15.5%, respectively. The RSD results (Table 2) of 784 points revealing clearly that screen printed Ag patterns are highly reproducible for various chemicals determination. Moreover, SERS intensity mapping of R6G (Fig. S2 in Support Information) and MG (Fig. S3 in *Support Information*) at different wave numbers also show the distribution of the band vibration intensity that is well-distributed. This further demonstrates that our screen printed Ag patterns are uniform in a large area and capable of generating SERS signals with good reproducibility.

**Figure 7:**
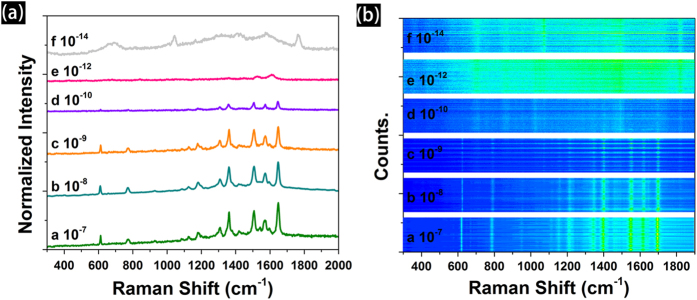
The SERS spectra (**a**) and reproducibility (**b** a randomly selected area, more than 120 points) of R6G with different concentration from 1 × 10^–7^ to 1 × 10^–14^ M adsorbed on the screen printed Ag patterns.

Furthermore, the practical detection limit and reproducibility of the detection of R6G molecules have been analyzed using our screen printed Ag patterns. Figure 7a shows the SERS spectra of R6G with different concentration from 1 × 10^–7^ to 1 × 10^–14^ M, the intensity and resolution of R6G signal is decreased with the decreasing concentration. Spectrum f (1 × 10^–14^ M) is same as the blank Raman spectrum of the printed Ag substrate which shows only a broad background with no detectable Raman signals of R6G (Figure S4 in *Support Information*). The partial characteristic peaks (1396 cm^−1^ and 1507 cm^−1^) of R6G still can be found in spectrum e. However, the spectrum resembles the features of R6G spectra, and thus the detection limit for R6G using our screen printed Ag substrate is higher than 1 × 10^−10^ M. Moreover, we use time evolution SERS spectra to monitor the signal output and study the reproducibility of the screen printed Ag patterns for R6G at different concentration. Figure 7b shows the Raman contour plots of recorded from a randomly selected area of the screen printed Ag pattern (102 points). Obviously, there are fluctuations on some band vibrations when the concentration of R6G reached at 1 × 10^–9^ M. The intensity and reproducibility of the band vibrations are improved if the concentration of R6G is gradually increased. Clearly, the spectrum of R6G with the concentration of 1 × 10^–7^ and 1 × 10^–8^ M is stable over time with high reproducibility. The above results demonstrate that the fluctuations depend on the concentration of R6G. Additionally, there are reports on coupled NPs improving the detection abilities by the generation of large number of hot-spots^21^,^22^,^23^,^24^. When the concentration of R6G is 10−^10^ M, the estimated number of molecules is about 5 (the estimation is provided at the *Support Information*). Above results illustrate that as-printed Ag substrate possesses high-sensitivity for the detection of R6G molecules. Indeed, R6G molecules can also adsorbed on the surface of Ag NPs and gaps between neighboring Ag NPs during the evaporation of ethanol solution^25^. Thus, Raman signals are detected efficiently by the hot-spots of as-printed Ag patterns.

**Figure 8:**
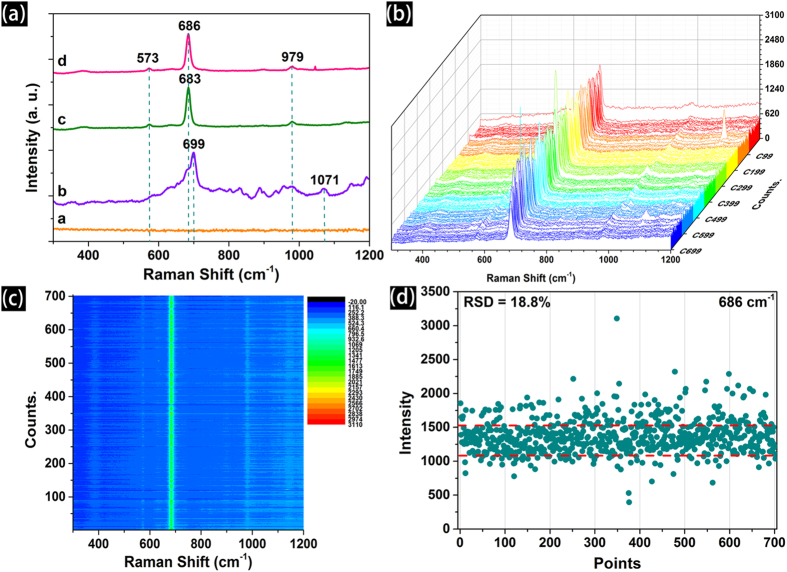
(**a**) The SERS performance of screen printed Ag patterns for melamine contaminated fresh milk (curve a: a commercial fresh milk; curve b: pure melamine in ethanol; curve c: 1 × 10^−2^ M of MA in milk; curve d: 1 × 10^−4^ M of MA in milk); (**b**) The waterfall plot of SERS spectra (integration time was 2 s, step width is 2 μm); (**c**) Raman contour plot shows both the intensity and reproducibility of 702 signals (randomly selected an area) from the printed patterns for MA contaminated fresh milk (1 × 10^−4^ M); (**d**) The intensity distributions of the main Raman vibrations of MA (at 686 cm^−1^) in 702 spots (RSD indicates).

Melamine (2,4,6-triamino-1,3,5-triazine) is known for milk powder adulteration in China, which caused lives and severe economic loss for the dairy industry. Hence, we study the SERS performance of the screen printed Ag patterns for the melamine contaminated fresh milk (with no pretreatment process), and the results are shown in Fig. 8. As shown in Fig. 8a, no signal is detected in pure fresh milk (curve a), and two peaks located at ∼699 cm^−1^ and ∼1071 cm^−1^ in pure melamine (curve b), which could be assigned to the ring breathing and ring deformation, respectively. When melamine is mixed in the milk (curve c and d), the two peaks at ∼573 cm^−1^ and ∼979 cm^−1^, could be seen and assigned to the δ (NCN)+τ (NH_2_) and δ (CNC) + δ (NCN), respectively (δ is bending vibration and τ is twisting vibration)^26^. In addition, two peaks at with different intensities were found at 683 cm^−1^ and 686 cm^−1^, respectively. Compare with the SERS spectrum of pure MA, the position is downshifted about 16 cm^−1^, which is caused by the different environment of MA in solution phase and in dried solid state^27^,^28^. Figure 8b show the SERS waterfall plots of MA collected from randomly selected area of the screen printed pattern with the concentration of 1 × 10^−4^ M, and an integration time of 2 s. The corresponding Raman contour plots show both the intensity and reproducibility of 702 signals as depicted in Fig. 8c. The results illustrate that an important feature of the screen printed Ag patterns is the excellent reproducibility of the SERS signals. Each spot shows distinctive Raman intensity, thus revealing excellent capability to enhance the Raman signals of the MA molecules in milk, a relatively complex system. The strong SERS signals demonstrate that screen printed Ag dot arrays possess high density “hot spots”, resulting in high reproducibility. As shown in Fig. 8d, the RSD of the Raman intensity of the 686 cm^−1^ (702 signals) is calculated, and the value is 18.8%, which further demonstrate the high reproducibility of the screen printed Ag patterns. The relatively low RSD variation of the SERS band illustrates the screen printed Ag patterns can be as a promising substrate for high-sensitivity SERS detection^29^. And SERS intensity maps of MA (Fig. S5 in Support Information) at 686 cm^−1^ also shows the uniform distribution of the Raman peaks intensities, and which reveal that the screen printed Ag patterns have the capacity of producing reproducible and large-area homogeneous SERS signal for a complex system. Indeed, the fresh milk is a mixture of various components and generates a solid milk film once the milk evaporates from the surface of SERS substrate. The solid milk film the SERS sensitivity of the as-printed Ag layer^36^.

## Conclusions

In summary, a facile approach to fabricate low-cost and highly reproducible SERS arrays using screen printing Ag NPs on flexible PET substrates has been explored. The printed Ag patterns can be used to detect various chemicals and show a well-defined output signals for R6G, MG, ATP, MNB and BBR. The spot-to-spot Raman spectra of R6G and MG at 1 × 10^−6^ M show significant Raman detection reproducibility in a large area, and the RSD results are less than 20%. Moreover, the detection limit for R6G on the screen printed Ag substrate is higher than 1 × 10^−10^ M. In addition, the screen printed Ag patterns is used to detect the melamine in fresh milk without pretreatment. Clearly, the current screen-printed approach offers a facile strategy to prepare large-scale, practical SERS substrates with high sensitivity and significant reproducibility, which would facilitate and improve the development of low-cost, disposable routine SERS detection platform.

## Author Contributions

W.W. designed the experiments. W.W., L.L. and S.Y. carried out the experiments and and analyzed the data. Z.D., J.L., L.Z. and X.X. assisted in Raman experiments. W.W., C.J. and V.A.L.R. supervised the project and finalized the manuscript. All authors contributed to the discussion of the results as well as to the writing of the manuscript.

## Additional Information

**How to cite this article**: Wu, W. *et al.* Low-Cost, Disposable, Flexible and Highly Reproducible Screen Printed SERS Substrates for the Detection of Various Chemicals. *Sci. Rep.* **5**, 10208; doi: 10.1038/srep10208 (2015).

## Supplementary Material

Supplementary Information

## Figures and Tables

**Table 1:** Vibrational modes of SERS peaks in different chemicals.

**Table 2:** RSD values with predominant peaks frequency of R6G (1 × 10^−6^ M) and MG (1 × 10^−6^ M).

**Analyte**	**R6G**				**MG**			
Predominant peaks (cm^−1^)	1362	1507	1574	1649	1362	1396	1592	1616
RSD value (%)	12.1	11.3	11.4	11.7	14.9	15.9	16.6	15.5
