# Supplementary material for: Low-Cost, Disposable, Flexible and Highly Reproducible Screen Printed SERS Substrates for the Detection of Various Chemicals
Source: Sci Rep. 2015 May 14;5:10208. doi: 10.1038/srep10208 (PMC4431467; doi:10.1038/srep10208)
Supplement: Supplementary Information [file srep10208-s1.doc]

**Support Information**

**Low-Cost, Disposable, Flexible and Highly Reproducible Screen Printed SERS Substrates for the Detection of Various Chemicals**

Wei Wu^1 ,2^*, Li Liu^1^, Zhigao Dai^3^, Juhua Liu^1^, Shuanglei Yang^4^, Li Zhou^2^, Xiangheng Xiao^3^, Changzhong Jiang^3^, Vellaisamy. A. L. Roy^2^*

^1^ Laboratory of Printable Functional Nanomaterials and Printed Electronics, School of Printing and Packaging, Wuhan University, Wuhan 430072, P. R. China

^2^ Department of Physics and Materials Science, City University of Hong Kong, Tat Chee Avenue, Kowloon Tong, Hong Kong SAR, P. R. China

^3^ Key Laboratory of Artificial Micro- and Nano-structures of Ministry of Education, School of Physics and Technology, Wuhan University, Wuhan 430072, P. R. China

^4^ State Key Laboratory for Powder Metallurgy, Central South University, Changsha 410083, P. R. China

Corresponding authors: [weiwu@whu.edu.cn](mailto:weiwu@whu.edu.cn) (W. Wu) and [val.roy@cityu.edu.hk](mailto:val.roy@cityu.edu.hk) (V. A. L. Roy)


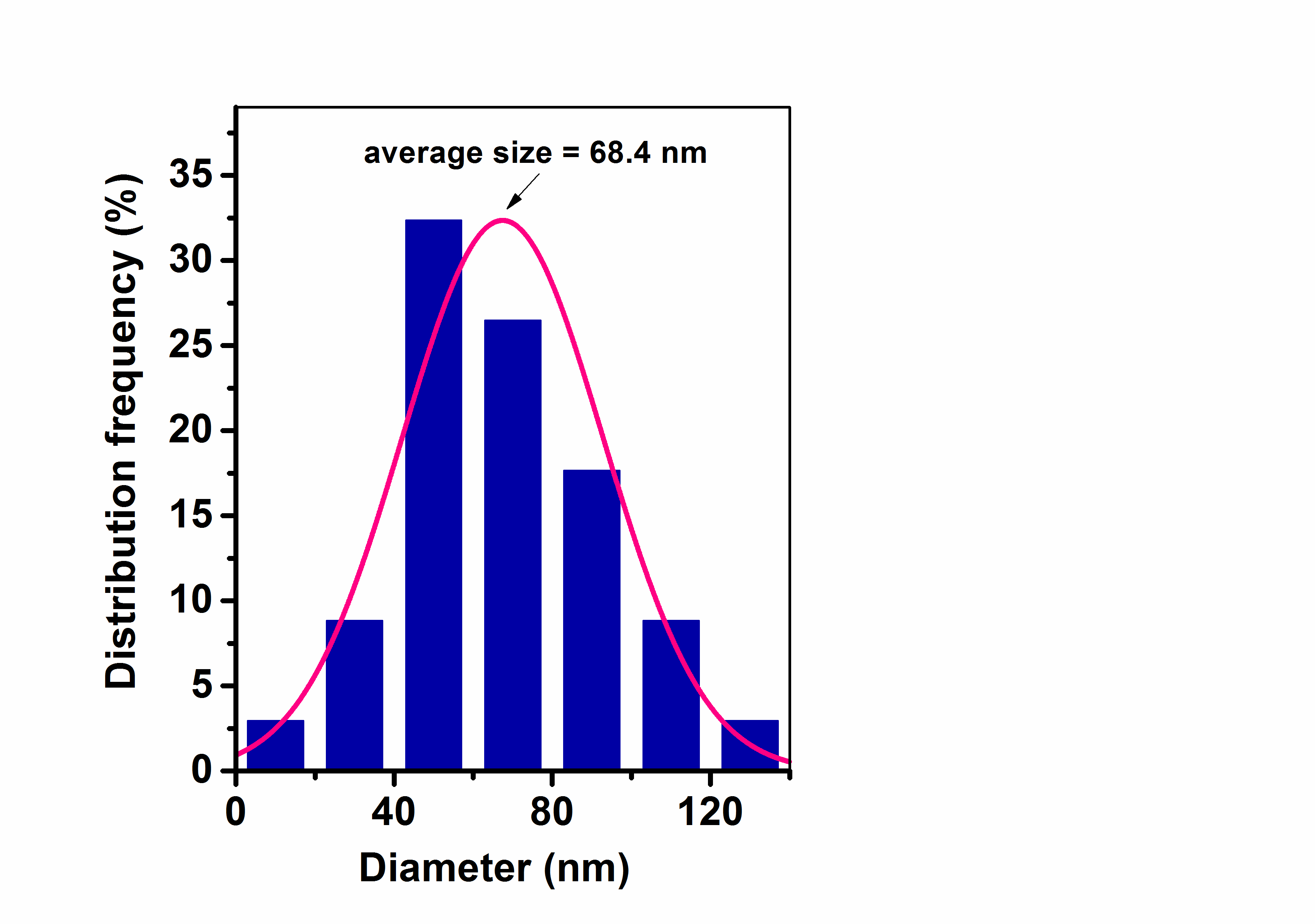


**Figure S1** Size histograms of the length and outer diameter of as-prepared Ag nanoparticles.


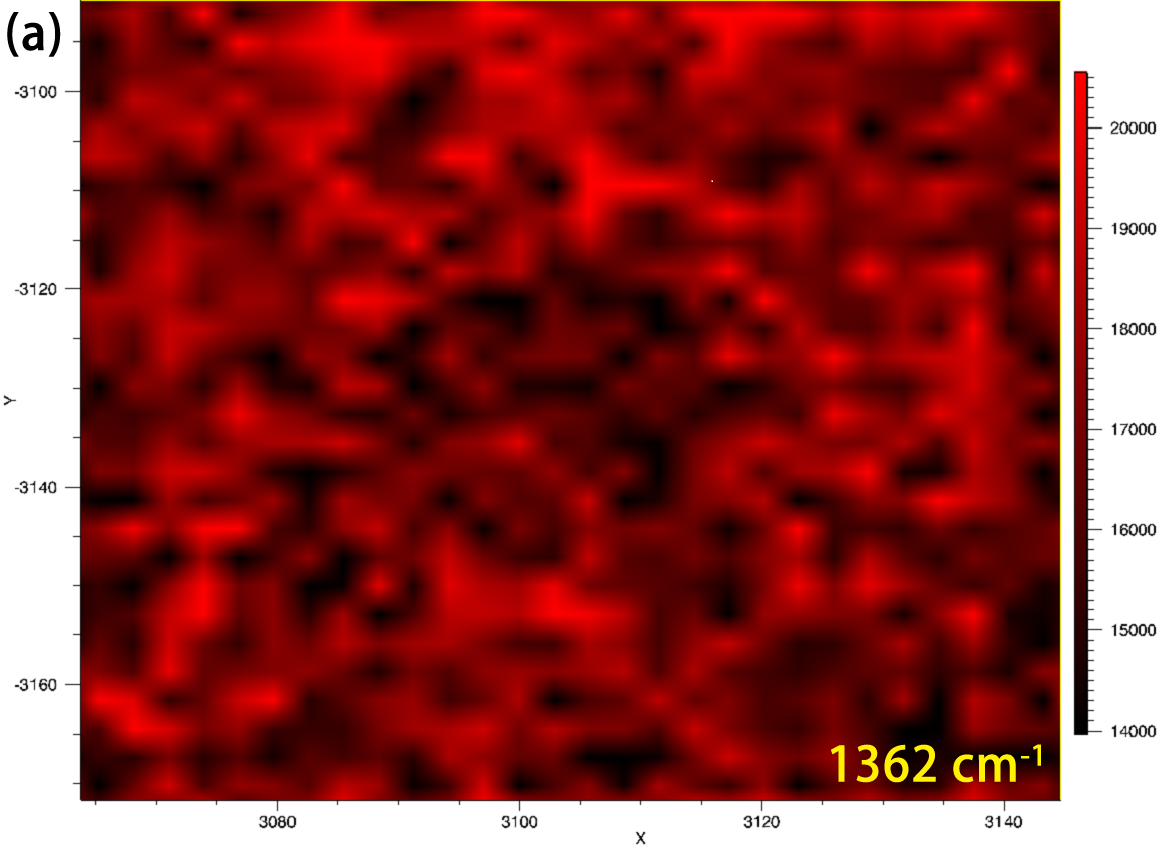

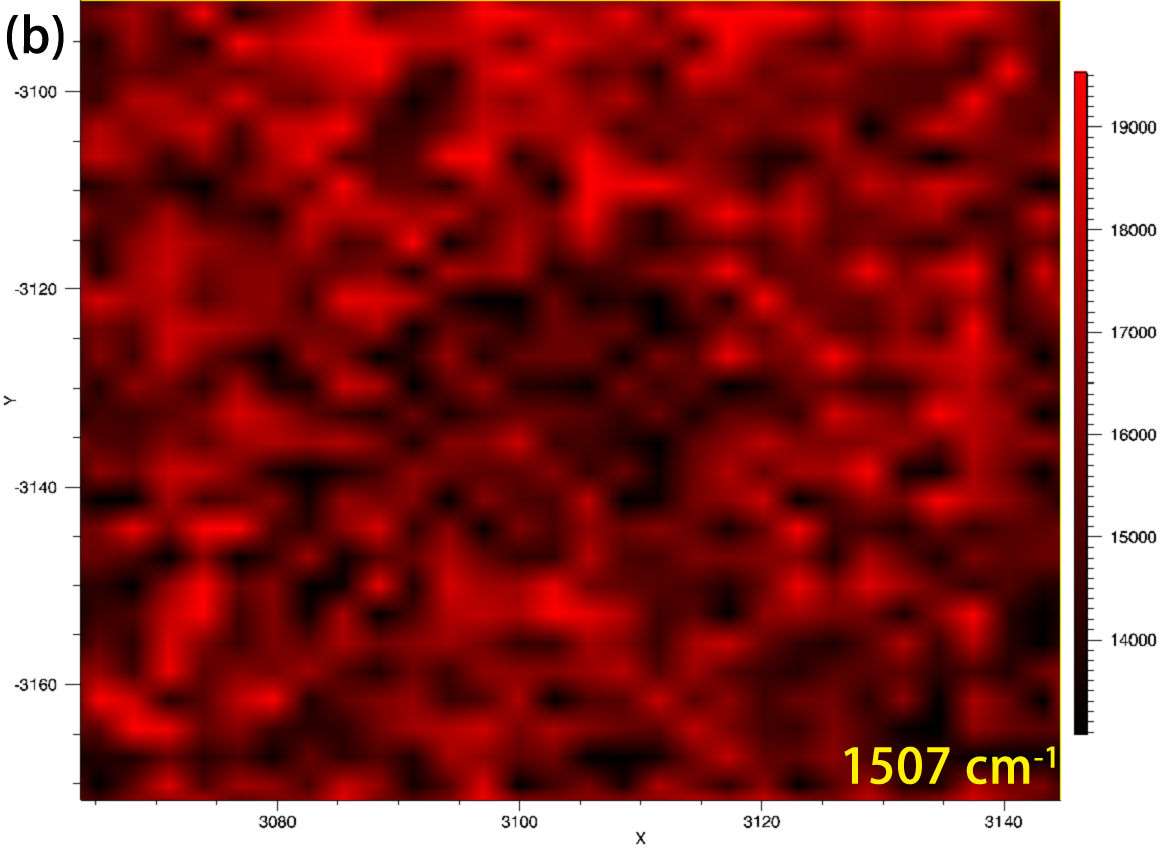

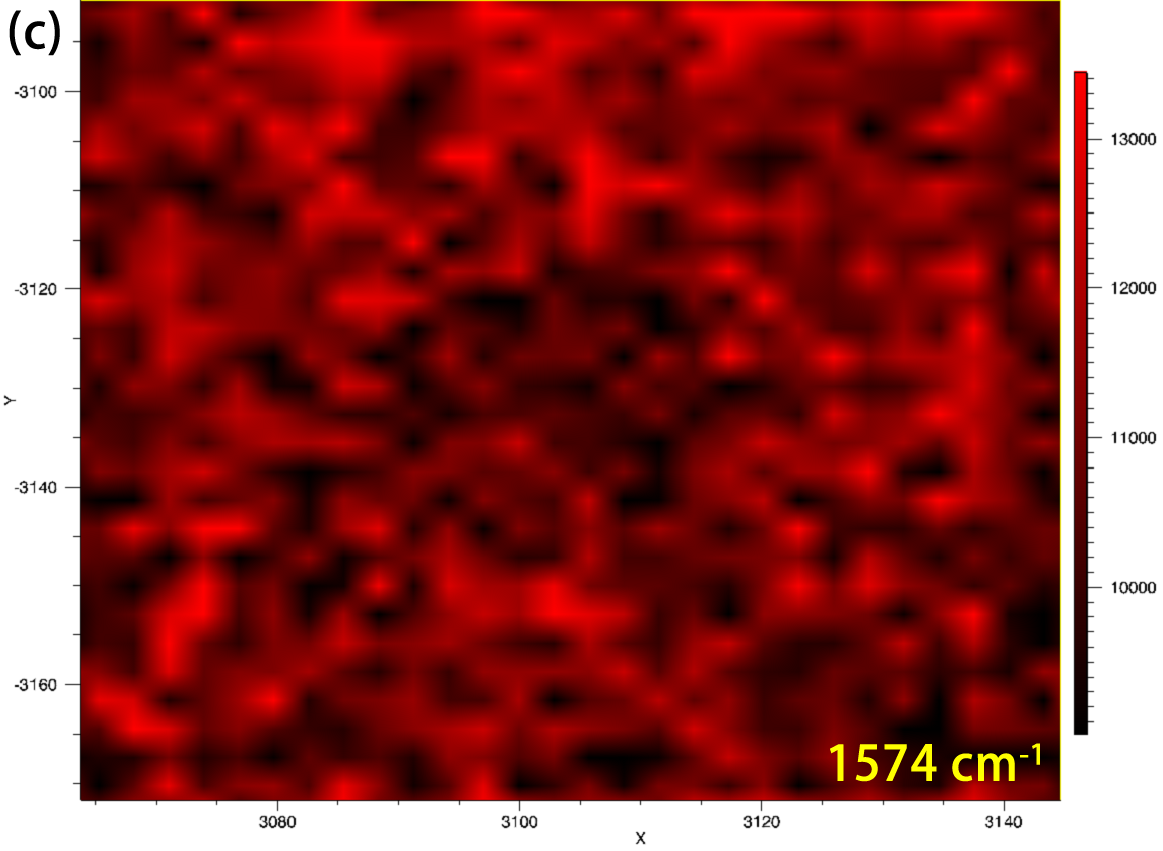

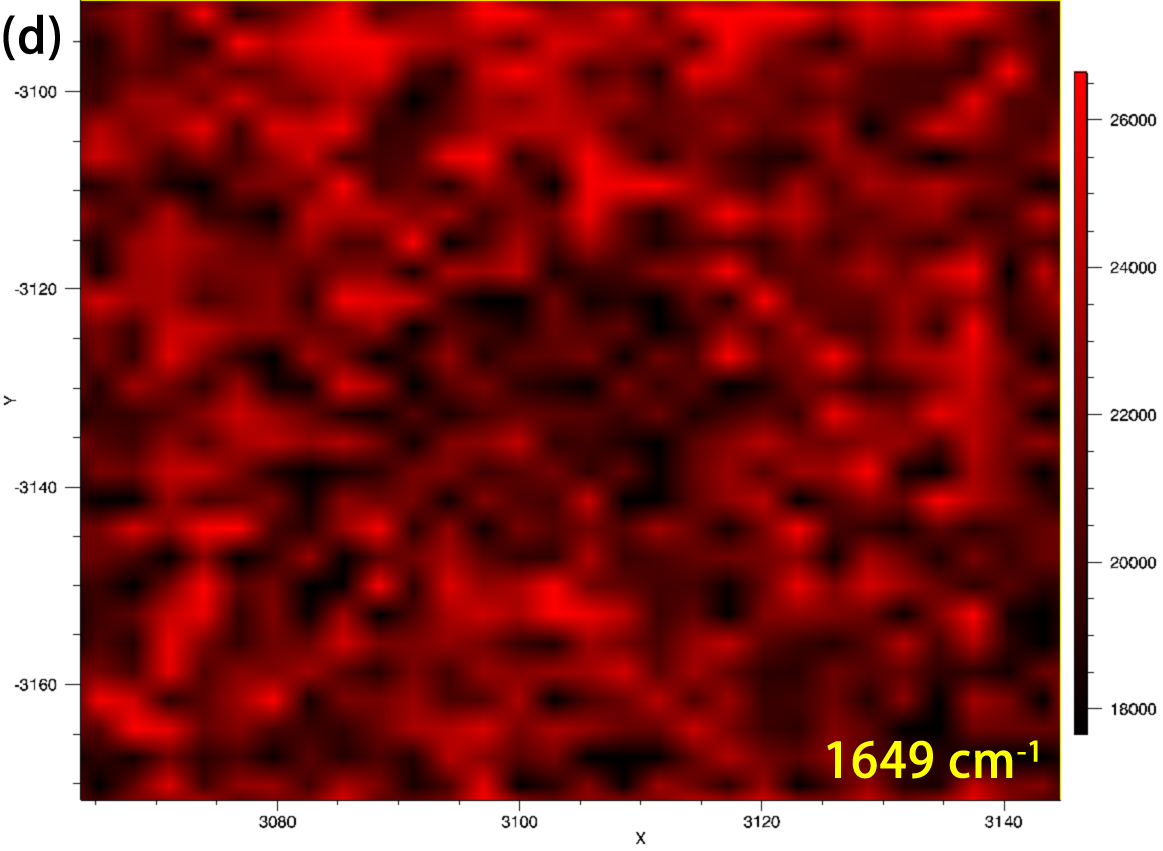


**Figure S2** SERS intensity maps of R6G at 1362 cm^-1^ (a), 1507 cm^-1^ (b), 1574 cm^-1^ (c) and 1649 cm^-1^ (d).


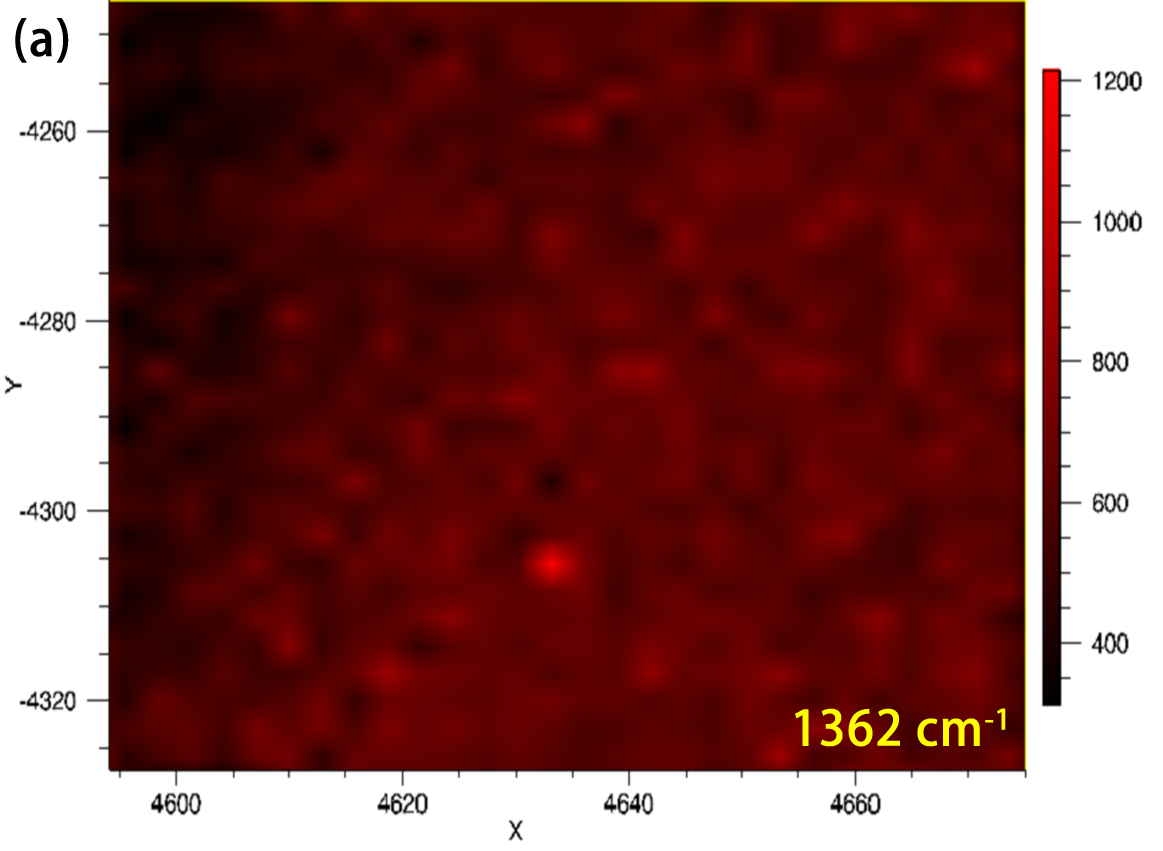

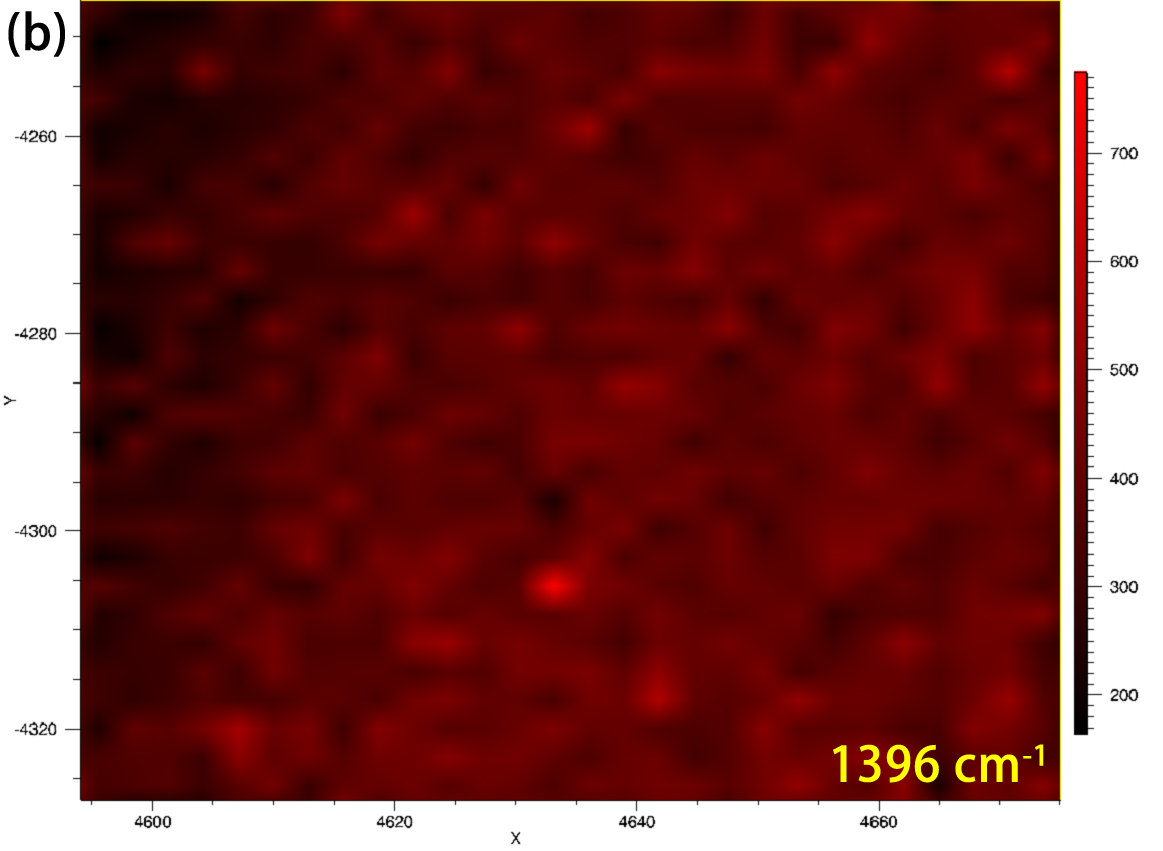

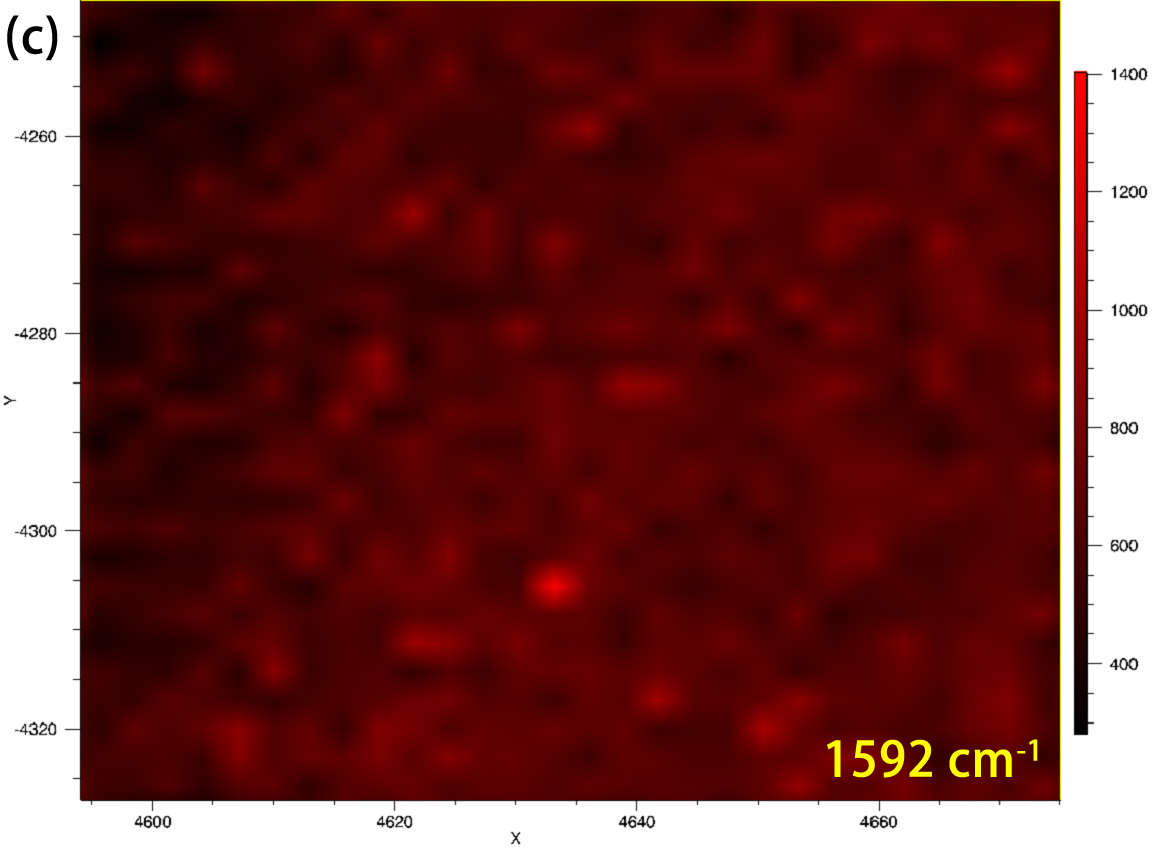

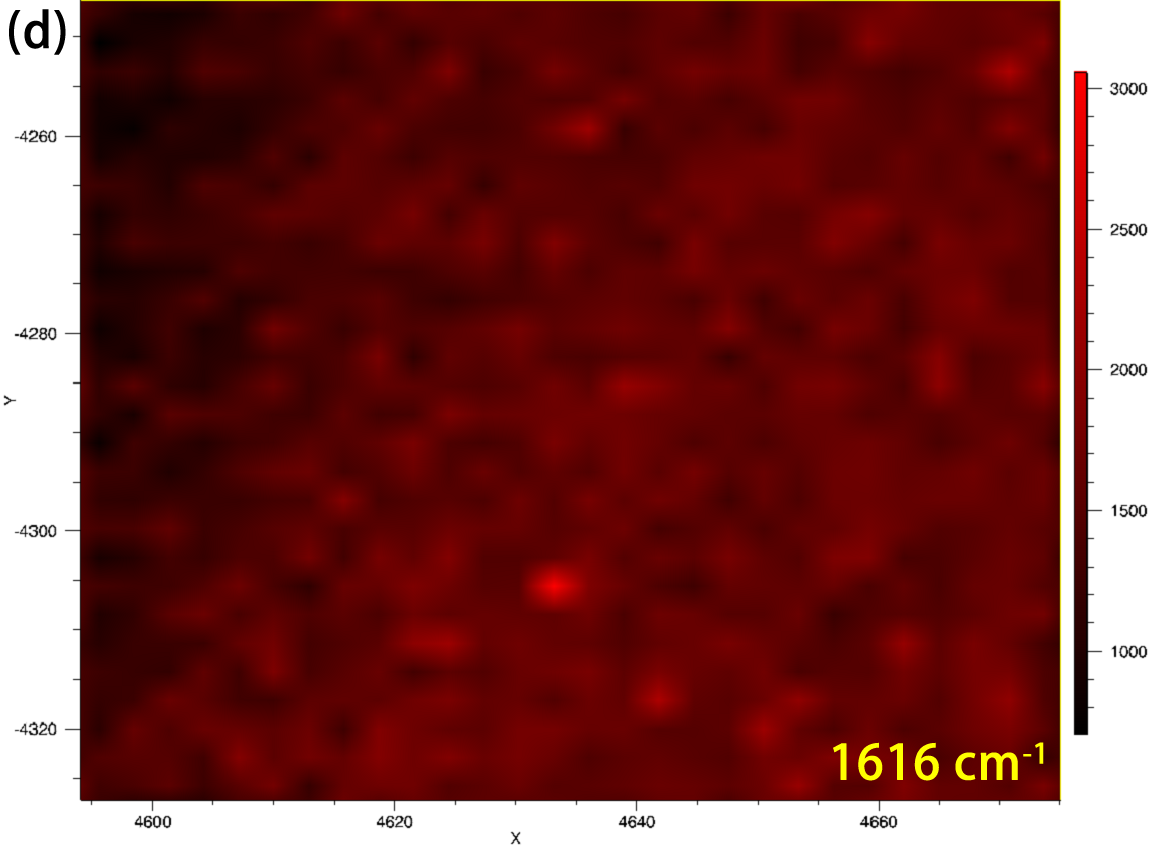


**Figure S3** SERS intensity maps of MG at 1362 cm^-1^ (a), 1396 cm^-1^ (b), 1592 cm^-1^ (c) and 1616 cm^-1^ (d).


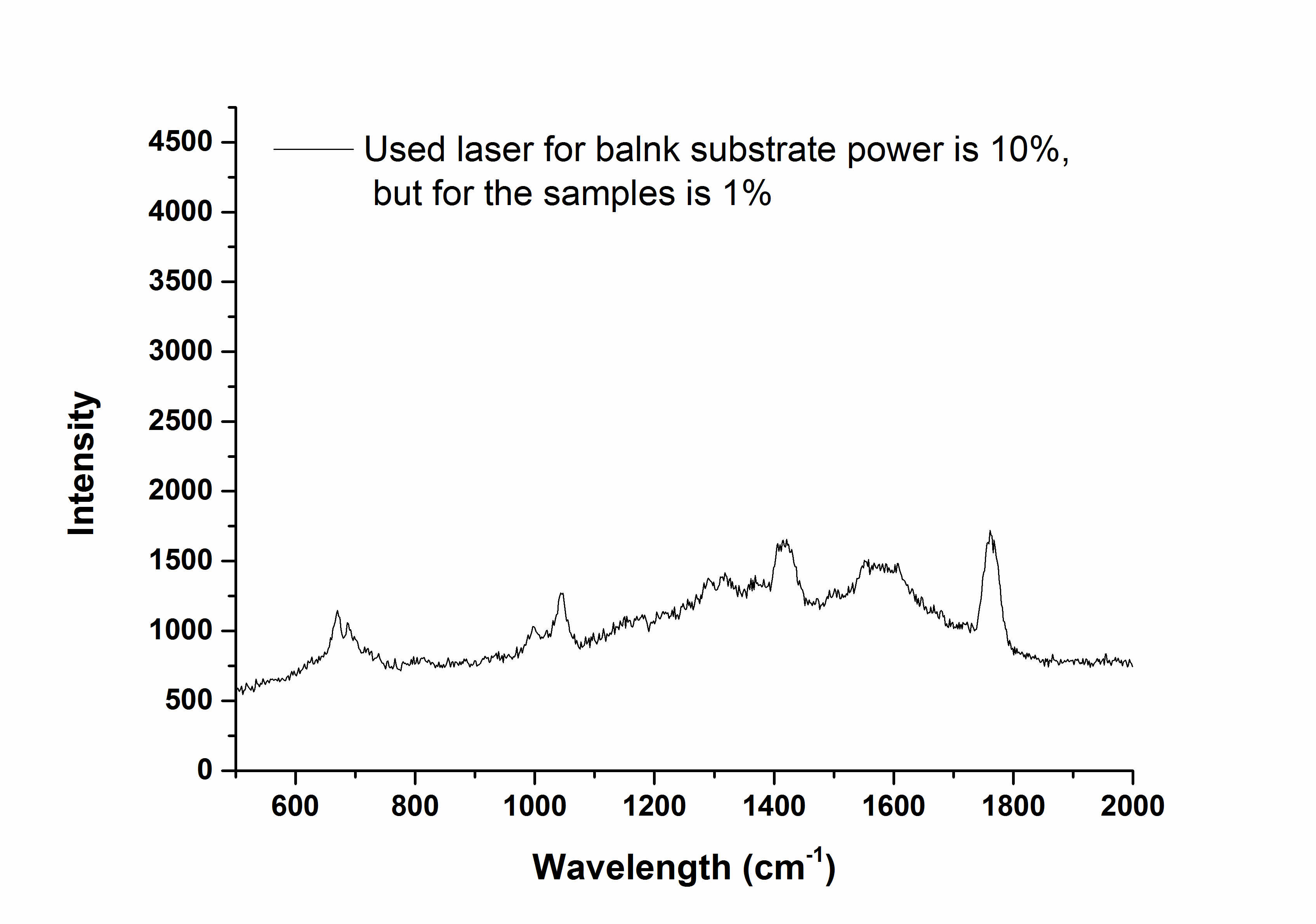


**Figure S4** The Raman spectra of the blank screen printed Ag substrate.

**
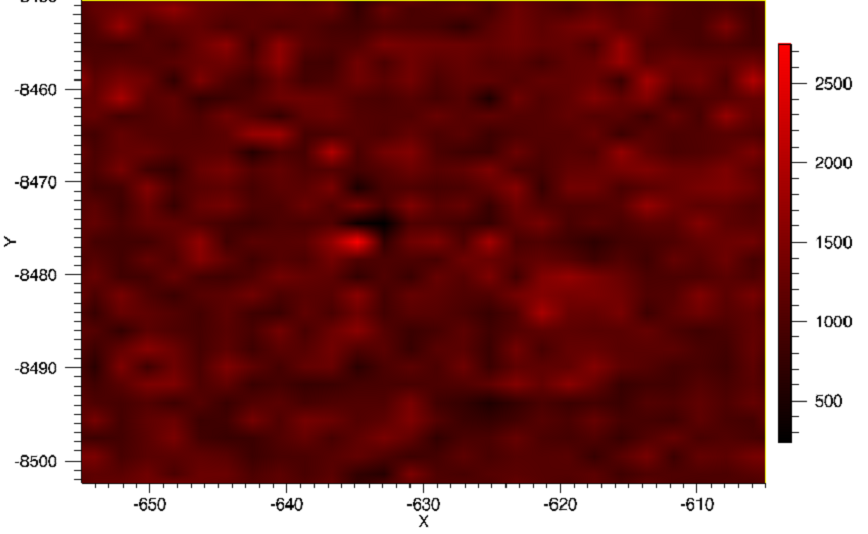
**

**Figure S5** SERS intensity maps of MA at 686 cm^-1^.

Molecules calculation process:

The diameter of laser spot size is around 700 nm at 100x magnification (D = 1.22λ/NA, λ is laser wavelength, and NA is numerical aperture, NA = 0.9), and hence the spot area (S_spot_) is about 7.7 × 10^-13^ m^2^. The number of molecules can be calculated by [(V_R6G_ × C_R6G_ × N_A_)/S_TD_] × S_spot_, where S_TD_ is the total dropped size is 10^-4^ m^2^ (1 mm × 1 mm), N_A_ is Avogadro constant, V_R6G_ is the dropped volume and the value is 10^-5^ L, when the concentration of R6G is 10^-10^ mol/L, the number of molecules is about 5.
